# Functional and evolutionary implications from the molecular characterization of five spermatophore CHH/MIH/GIH genes in the shrimp *Fenneropenaeus merguiensis*

**DOI:** 10.1371/journal.pone.0193375

**Published:** 2018-03-19

**Authors:** LiLi Shi, Bin Li, Ting Ting Zhou, Wei Wang, Siuming F. Chan

**Affiliations:** Fisheries College, Guangdong Ocean University, Zhanjiang, PR China; Biocenter, Universität Würzburg, GERMANY

## Abstract

The recent use of RNA-Seq to study the transcriptomes of different species has helped identify a large number of new genes from different non-model organisms. In this study, five distinctive transcripts encoding for neuropeptide members of the CHH/MIH/GIH family have been identified from the spermatophore transcriptome of the shrimp *Fenneropenaeus merguiensis*. The size of these transcripts ranged from 531 bp to 1771 bp. Four transcripts encoded different CHH-family subtype I members, and one transcript encoded a subtype II member. RT-PCR and RACE approaches have confirmed the expression of these genes in males. The low degree of amino acid sequence identity among these neuropeptides suggests that they may have different specific function(s). Results from a phylogenetic tree analysis indicated that these neuropeptides were likely derived from a common ancestor gene resulting from mutation and gene duplication. These CHH-family members could be grouped into distinct clusters, indicating a strong structural/functional relationship among these neuropeptides. Eyestalk removal caused a significant increase in the expression of transcript 32710 but decreases in expression for transcript 28020. These findings suggest the possible regulation of these genes by eyestalk factor(s). In summary, the results of this study would justify a re-evaluation of the more generalized and pleiotropic functions of these neuropeptides. This study also represents the first report on the cloning/identification of five CHH family neuropeptides in a non-neuronal tissue from a single crustacean species.

## Introduction

The X-organ sinus gland complex of the crustacean eyestalk is known to produce many important neuropeptides that control important, diversified physiological processes [1–3]. Although the red-pigment concentrating hormone (RPCH) and the pigment concentrating hormone of the chromatotrophins were the first group of neuropeptides to be fully characterized [4], the crustacean hyperglycemic hormone (CHH), molt inhibiting hormone (MIH) and gonad inhibiting hormone (i.e., CHH/MIH/GIH neuropeptides) have attracted much research attentions [5–7] because of their potential use in improving the aquaculture of many economically valuable species. They are the most popular group of crustacean neuropeptides examined so far. At present, a large amount of research efforts has focused on the study of CHH family neuropeptides. However, we still know very little about this group of important neuropeptides. One of the reasons is the lack of concerted and focused study on this group of neuropeptides in a single species. Furthermore, there are still many unidentified members in a single species. Due to their economic importance, there are much information on the CHH neuropeptides in shrimps [5, 8, 9]. Because of the structural similarity of the CHH-family members, they are often known to have overlapping functions. For example, in the crayfish *Procambarus clarkii*, CHH is involved in both regulation of circulating glucose levels and inhibition of ecdysteroid synthesis [10]. The molt inhibiting hormone of the shrimp *Metapenaeus ensis* (MeMIH-B) has also been shown to have gonad-stimulating properties [6, 11]. At present, there are >100 eyestalk neuropeptide cDNAs identified from different crustaceans. However, it is confusing that the current naming and identification of CHH members have been restricted to only a few that depend mainly on their similarity with other species. Most of the studies focus on the molecular characterization of cDNA, but only a few functional analyses (<10% of those identified cDNA) have been performed for purified neuropeptides or recombinant protein of these cDNAs. Therefore, functional annotation of most of these cDNAs are inaccurate as the naming of these neuropeptides in most species is based on sequence similarity. Without identifying all the members of this gene family, it would certainly lead to mislabeling of gene names.

Initially, the expression of CHH family neuropeptide was thought to be restricted to the eyestalks. This concept has changed since some CHH family members are also found to be expressed in other neuronal tissues such as the thoracic ganglion and subesophageal ganglion [12]. Recent evidence has shown that other non-neuronal tissues such as the pericardial organ [13, 14], gill [15], spermatophore [16] and intestine [17, 18] can also express these neuropeptides. Furthermore, CHH family neuropeptides have been shown to act in metabolism and osmoregulation [19] as well as in immune defense [20]. With the recent use of transcriptome sequencing and molecular cloning techniques, many more CHH neuropeptide cDNAs from a single species of crustacean have been identified. For example, in the transcriptomic study of the freshwater shrimp *Macrobrachium rosenbergii*, more than 3 CHH transcripts have been identified in the eyestalk [21].

The banana shrimp *Fenneropenaeus merguiensis* is the major marine shrimp species harvested in Southern China. Recently, there has been an increase in research interest in this local species since aquaculture production from the exotic species (*Litopenaeus vannamei*) has decreased. However, much of the physiology, reproduction and culture techniques for this species is not well studied. Unlike most other cultured penaeid shrimps, female *F*. *merguiensis* can mature easily in captive conditions. Here, we have initiated a banana shrimp breeding technique development program and have studied the male reproductive biology of this species. One aim of our research program is to identify important sex-related genes in the male reproductive system that might be useful in predicting sperm quality. To our surprise, we have identified five CHH-family transcripts in the spermatophore transcriptome. The following is our report on the molecular characterization of these CHH neuropeptides. We have utilized a comparative evolutionary approach to characterize the transcripts identified in an attempt to correlate their functions in the control of glucose, molting, and reproduction.

## Material and method

### Animals

All animal experiments reported in this study were conducted in compliance with the Guideline for the Use of Experimental Animal of Guangdong Ocean University. Wild-caught adult shrimp were purchased from a local seafood market and juveniles were obtained from the shrimp genetic breeding facility of Guangdong Ocean University. They were acclimated to laboratory conditions for 1week before the start of the experiment.

### RNA extraction and transcriptome sequencing

Adult males were divided into two groups of ten each. The first group was unilateral eyestalk ablated and the second group was an intact control. Three to four days after eyestalk ablation, the spermatophores from the intact and unilateral eyestalk ablated males were dissected and the tissue was initially stored at -80°C until RNA preparation. Total RNA was prepared using an RNA extraction kit (Sangon, China). RNA libraries were further constructed before being sequenced as 150 bp paired-end reads on the Illumina HiSeq 2500 platform. The transcriptome dataset was obtained for the mining of genes described in this study.

### Bioinformatic analysis of the spermatophore CHH family neuropeptides

CHH family transcripts from the spermatophore transcriptome were analyzed using the BLASTN, BLASTX, and BLASTP programs of the GenBank database (https://www.ncbi.nlm.nih.gov/genbank). The best matched shrimp sequence from the search were retrieved from GenBank for the alignment and phylogenetic study described in this paper. Prediction of the signal peptide was performed using the SignalP program (http://www.cbs.dtu.dk/services/SignalP/). Amino acid sequences for the CHH family members from different shrimps, crabs and lobsters used in this study were retrieved from the GenBank database.

CHH family type I sequences include the shrimp *Penaeus monodon* (PmSGP-I: #AF104386, PmSGP-II: #AF104387, PmSGP-III: #AF104388, PmSGP-IV: #AF104389, PmSGP-V: #AF104390, PmCHH1: GQ221085, PmCHH2: AY346397, PmCHH3: AY346380, PmGIH: #KT906363), *F*. *chinensis* (FcCHH1: #JQ012758, FcCHH2: #JQ012759), *Litopenaeus vannamei* (LvCHH: #KU174974, #JX856151), and *Metapenaeus ensis* (MeMC: #AB461933, MeMA: #AF076276).

CHH family type II sequences include the shrimps *P*. *monodon* (PmMIH1: #AY496451, PmMIH2: #AY496455, PmSGPC1: #AB054187, PmSGPC2: #AB054788), *F*. *chinensis* (FcMIH: #AF312977), *M*. *ensis* (MeeMB: #AF294648), *L*. *vannamei* (LivMIH1: #AY425616, LivMIH2: #AY425616, LivGIH: #KF879913), *Marsupenaeus japonicus* MjMIH-B #AB162448; *Macrobrachium rosenbergii* (MrSGP-A: #AF432347), *Macrobrachium niponensis* (MnMIH: #KF878973), *Palaemon carinicauda* GIH (PcGIH: #KM210513), the lobster *Homarus gammarus* (HgVIH: #DQ181793), the crab *Cancer pagurus* (CpMOIH1: #AJ245378, CpMOIH2: #AJ245379), and the locust *Schistocerca gregaria* ion transport protein (SgITP: U36920).

For the alignment and sequence homology study, the pre-propeptide or mature peptide region of the *F*. *merguiensis* CHH family neuropeptides were aligned with CHH family members from other shrimps using ClustalW IX 2.0.12 (genome.jp/tools/clustalw). For phylogenetic tree construction and analysis, selected sequences in this study were first aligned with ClustalX and the aligned sequences were used to construct the phylogenetic tree using the neighboring method with the program Mega 6.0 [22] with bootstrap replication of N = 1000.

### Expression study of the eyestalk neuropeptides

Total RNA from different tissues were prepared for gene expression studies. To analyze the expression of these neuropeptide genes in spermatophores of the male shrimp after unilateral eyestalk ablation, quantitative real time PCR (qPCR) was performed with elongation factor (EF-1) gene used as internal control. To ensure specific amplification, genomic DNA was removed by DNAse I treatment during first strand cDNA synthesis step and the primers spanning 2different exons of each spermatophore transcript were used (S1 Table). One microliter of the diluted cDNA reaction mix (1:25X dilution) was used as template in a 25 μl PCR volume. Reactions were performed using a CYBR green 2X qPCR kit (Takara Bio Inc.) and a CFX Connect Real Time System (Bio-Rad Laboratories, CA). The PCR protocol consisted of 95 ^o^C for 1 min, 40 cycles of 95 ^o^C for 30 s, 58 ^o^C for 25 s and 72 ^o^C for 25 s. Both β-actin and the elongation factor (EF-1) genes were used as internal control genes. However, as the expression of EF-1 was invariantly stable, therefore we have used EF-1 gene as internal control genes to normalize the spermatophore CHH-family member expression after serial dilution and the slopes of the curves were obtained. The experiments were performed in triplicate. The qPCR data were analyzed using MX Pro-Mx3000P Multi-plex Quantitative PCR system Software (LightCycler480, Agilent) and the relative expression ratio (R) of mRNA was calculated according to the formula 2 -△^2^Ct. The melting curve analysis was performed to confirm the quality of the qPCR. The qPCR results were measured with the 2 -△^2^Ct methods. The data were normalized for each gene against those obtained for the elongation factor. The results are presented as means with standard deviations of fold increase/decrease from six shrimps at each data point. The transcript level of each CHH-family gene was compared to the intact and unilateral eyestalk ablated shrimp by two-way ANOVA, and the level of significance was set at P < 0.05 for all analyses. The statistical software SPSS was used to analyze all data collected and controls.

#### Eyestalk ablation experiment

Shrimp were divided into two groups of ten each. Unilateral eyestalk ablation was performed for 1group using a pair of hot forceps. The shrimp were then returned to normal culture conditions with the control (or untreated group). Five days after the eyestalk removal, the shrimp were molt staged by pleopod setogenesis and total RNA was prepared from the spermatophore (N = 6) using the RNA extraction kit and the qPCR analysis as described above.

## Results

**Fig 1:**
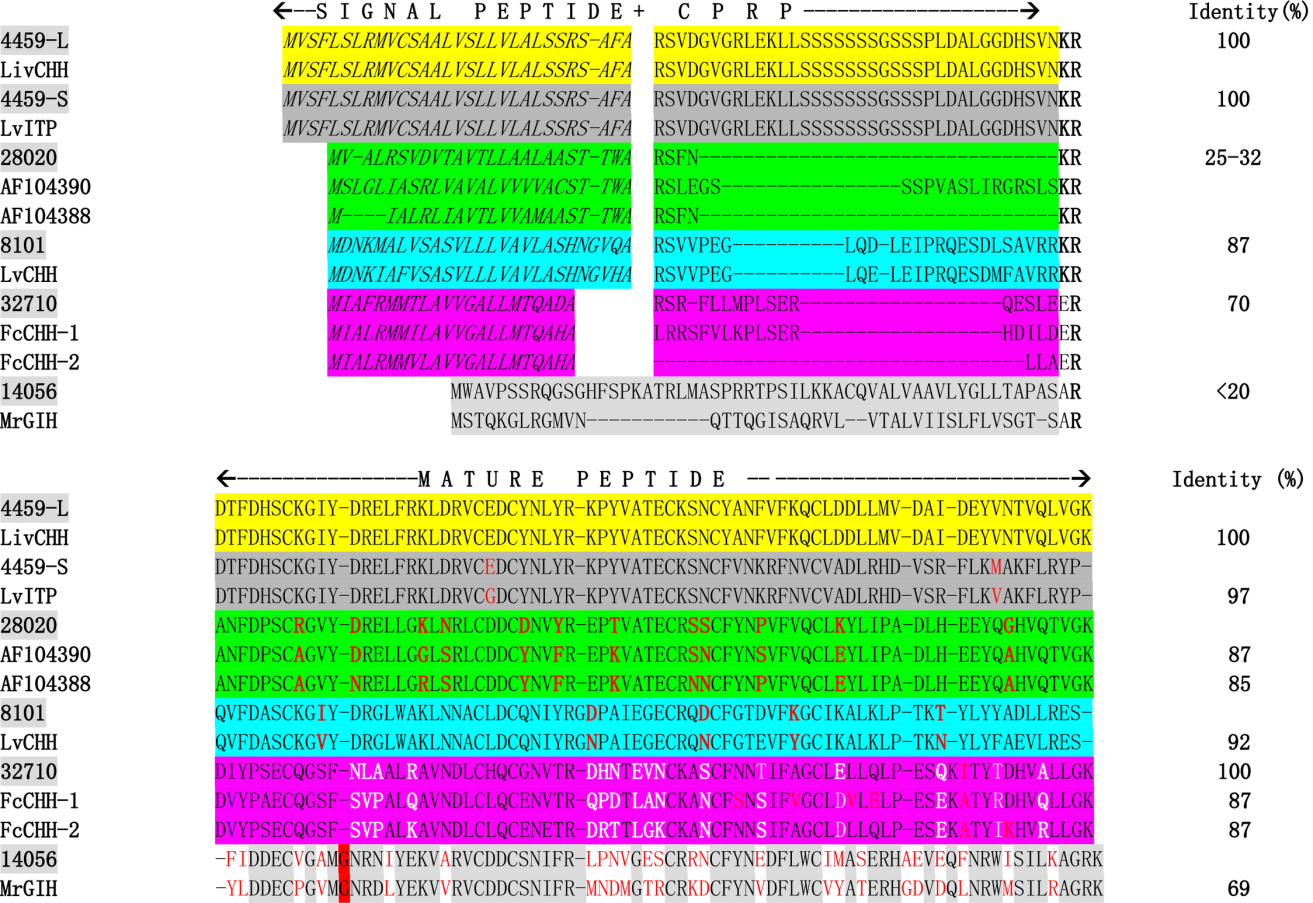
Alignment of the spermatophore CHHs with homologous sequence from different shrimps. The same horizontal colors indicate homologues that share a much higher degrees of amino acid identity in the mature peptide region. The predicted signal peptide of each member is in bold letters. The potential cleavage site is shown (in black bold letters) is the location to produce the mature peptide (i.e. KR and ER). No signal peptide was predicted for translate product of transcript 14056 and MrGIH and the glycine at position 11 is highlight in red. The identity (%) on the left of the table indicated the overall amino acid identity of the mature peptides between members of the same color horizontal block. Un-identical amino acid of the aligned sequence within the same horizontal color blocks are indicated by the bolded white or red letters.

Five transcripts from the spermatophore transcriptome of *F*. *merguiensis* were identified to encode for different eyestalk CHH/MIH/GIH family neuropeptide members (Figs 1 and S1 and Table 1). Sequences of these transcripts were searched for homology using the GenBank BLASTX or BLASTP program to obtain the most updated data from the database. In the analysis, we focused on searching for shrimp sequences that shared the highest identity with the translated sequences of these five transcripts.Transcript CL4459 was 1771 bp in size. It encoded a novel type I CHH neuropeptide (i.e., 4459-S) with 141 amino acid residues (Table 1). The deduced protein can be divided into a signal peptide region (i.e., amino acid residues 1–30), followed by a short CHH-related precursor peptide sequence (CPRP) with high serine (S) content (Table 2). The mature peptide is 73 amino acids in size and is produced by cleavage from the dibasic cleavage KR site (Fig 1). BLASTX search results indicated that the pre-propeptide sequence shared a high degree of similarity to the CHH of the shrimp *L*. *vannamei* (GenBank #AFV95080). Further analysis of the transcript revealed that a different reading frame of the cDNA encoded a fragment of amino acid sequence highly homologous to the C-terminal end of the shrimp *L*. *vannamei* LvITP (GenBank #AAN86055). The result suggest that the gene for transcript 4459 is similar to that of *L*. *vannamei* which is known to produce two different transcripts [15]. Alternative splicing of the 4459 pre-mRNA may produce a different transcript with an identical N-terminal end but a different C-terminal end of the peptide. To determine if the two different transcripts could be identified in a single shrimp, gene-specific primers were designed to amplify the two cDNAs resulted from the alternative splicing. RT-PCR results indicate that both transcripts could be detected in the gill, but only 1transcript was present in the eyestalk (i.e. transcript 4459-S) and spermatophore (i.e. transcript 4459-L) (S2 Fig). DNA sequence determination confirmed the existence of the alternative spliced form (i.e., 4459-L, Table 1, Fig 1). Alignment results indicate that the transcript shared 97.2% amino acid identity with LvITP of *L*. *vannamei* (Fig 1 & Table 1). In short, the gene for transcript 4459 can produce two different transcripts encoding two proteins that share identical N-terminal ends but have very different C-terminal ends.

**Table 1:** Gene ID / Transcript obtained from the spermatophore of *F*. *merguiensis*.

Gene ID	ORF (nt)	GenBank #	Nr-E value	Amino acid sequences	Species/Gene
4459-L	425	AFV95080	2.00E-74	MVCSAALVSLLVLALSSRSAFARSVDGVGRLEKLLSSSSSSSGSSSPLDALGGDHSVNKRDTFDHSCKGIYDRELFRKLDRVCEDCYNLYRKPYVATECKSNCFVNKRFNVCVADLRHDVSRFLKMAKFLRP	*L*.*vannamei/* CHH-like
44459-S	420	AAN86055	1.00E-69	MVCSAALVSLLVLALSSRSAFARSVDGVGRLEKLLSSSSSSSGSSSPLDALGGDHSVNKRDTFDHSCKGIYDRELFRKLDRVCEDCYNLYRKPYVATECKSNCYANFVFKQCLDDLLMVDAIDEYVNTVQLVGK*	*L*.*vannamei/* ITP
8101	569	AEI83867	1.00E-12	MDNKMALVSASVLLLVAVLASHNGVQARSVVPEGLQDLEIPRQESDLSAVRRRRQVFDASCKGIYDRGLWAKLNNACLDCQNIYRGDPAIEGECRQDCFGTDVFKGCIKALLPTKTYLYYADLLRES*	*E*.*pulchra/* CHH
28020	315	097385	2.00E-11	MVALRSVSVDETAVTLLAALAASTTWARSFNKRANFDPSCRGVYDRELLGKLNRLCDDCDNVYREPTVATECRS SCFYNPVFVQCLKYLIPADLHEEYQGHVQTVGK	*P*.*monodon/* CHH
32710	348	AFD32402	1.00E-39	MIAFRMMTLAVVGALLMTQADARSRFLLMPLSERQESLEERDIYPSECQGSFNLAALRAVNDLCHQCGNVTRDHNTEVNCKASCFNNTIFAGCLELLQLPESQKTTYTDHVALLGK	*F*.*chinensis/* CHH-like
14056	399	AEJ54622	5.00E-31	MWAVPSSRQGSGHFSPKATRLMASPRRTPSILKKACQVALVAAVLYGLLTAPASARFIDDECVGAMGNRNIYEKVARVCDDCSNIFRLPNVGESCRRNCFYNEDFLWCIMASERHAEVEQFNRWISILKAGRK*	*M*.*nipponensis/* GIH

One letter amino acid code is shown for each of the deduced peptide. Gene ID: names of gene in this study; ORF: open reading frame; the Genbank ID are sequences that matched best with the *F*. *merguiensis* CHH-family neuropeptide gene. Species/Gene name: the best matched shrimp sequence.

**Table 2:** SignalP analysis of the pre-pro-peptide sequence of the spermatophore derived CHH-family members.

Transcript	SP cleave position	D-value	SP (Y/N)	CPRP	MP (AA residues)
4459-S	31	0.849	Y SP = 31	31–68	73
4459-L	31	0.84	Y SP = 31	31–68	74
8101	27	0.788	Y SP = 27	28–54	74
28020	25	0.43	Y SP = 25	26–31	74
32710	22	0.747	Y SP = 22	23–41	75
14056	No	0.203	N	None	133
FmMIH	28	0.813	Y SP = 28	None	77
MrGIH	No	0.443	N	None	131

FmMIH is the deduced molt inhibiting hormone of *F*. *merguiensis* and MrGIH is the gonad inhibiting hormone of the fresh water shrimp *Macrobrachium rosenbergii*

Transcript CL8101 is 1519 bp in size and the longest ORF of this transcript is expected to encode for another novel CHH family member with 128 amino acid residues (Figs 1 and S1 and Table 1). Using SignalP prediction software, amino acids 1–27 are predicted to be the signal peptide and AA28-54 encoded for the CPRP. Cleaving of the propeptide at the KR site would produce the mature peptide with 74 amino acid (AA55–AA129). BLASTX search analysis results indicated that it is most similar (88% aa identity) to the recently discovered novel CHH reported in *L*. *vannamei* (GenBank #AEI83867) [13]. It showed a much lower sequence homology with other crustacean CHH family members from the GenBank database. The mature peptide lacked a glycine residue at position twelve. Thus, this placed this new member in the CHH-family subtype I category. Therefore, the deduced peptide from transcript 8101 appears to be more related to the subtype I CHH family neuropeptides. In *L*. *vannamei*, the CHH-like is expressed in the X-organ and optic nerve in the eyestalk, supraesophageal ganglion (SoG), gill, gut, pericardial cavity, as well as in the terminal ampoule or spermatophore and vas deferens of males [13, 16]. ClustalW alignment and phylogenetic tree analysis revealed that the signal peptide and mature peptide region of the two cDNAs share a high degree of amino acid sequence identity between the two species.

Transcript 28020 is 1690 bp in size with the longest ORF of 315 bp (Figs1 and S1 and Table 1). It is predicted to encode a type I CHH neuropeptide. The pre-propeptide consists of 105 amino acid residues (Table 1). SignalP 4.0 prediction results indicated that AA1-AA25 is the potential signal peptide and cleaving of the pro-peptide at the dibasic site (KR) resulted in a mature peptide with 74 amino acid residues (aa26-aa108) (Table 2). Results from the BLASTX search analysis, ClustalW sequence alignment and phylogenetic tree analysis revealed that it is most similar to the crustacean hyperglycemic hormone 3 of *P*. *monodon* (Pm-SGP-III. GenBank # O97385) [9], followed by the CHH precursor of *L*. *vannamei* (GenBank #BAM93361) [23]. It shares a moderate similarity with CHH members from other decapods including the lobster *Homarus americanus* CHH-B, (GenBank #ABA421800) [24], the hyperglycemic hormone of the crayfish *Astacus leptodactylus* (GenBank # AAX09331), the freshwater shrimp *Macrobrachium rosenbergii* (GenBank #AAL40915) [25], and the CHH of the hermit crab *Pagurus bernhardus* (ABE02191) [26]. However, unlike other CHH sequences, all of these CHH peptides lack the CPRP identified in the precursor sequences.

Despite the small transcript size (i.e., 531 bp), transcript 32710 was predicted to encode a full-length ORF of a type I neuropeptide precursor with 116 amino acid residues. The first 22 amino acids encoded for the signal peptide of the precursor and the remaining 75 amino acids encoded for the mature peptide. BLASTX search analysis results indicated that it was most similar to the CHH-like peptide of the shrimp *Fenneropenaeus chinensis* [16]. ClustalW sequence alignment results, however, revealed that the overall sequence similarity between the 2 molecules is quite low (i.e., 70–72%).

Transcript 14056 is the largest of the 5 CHH transcripts identified (S1 Fig) from the spermatophore transcriptome (i.e., 1763 bp). It is predicted to encode for a pre-propeptide with 133 amino acid residues (Table 1). No signal peptide was predicted by the SignalP prediction program (Table 2). It belongs to the CHH family subtype II neuropeptides with their distinctive features: 6conserved cysteine residues and an additional glycine residue at position 12 in the mature peptide. Therefore, this peptide represents the only type II CHH neuropeptide family member recorded in this species. Interestingly, BLASTX search analysis, ClustalW sequence alignment and phylogenetic analysis results indicated that this pre-propeptide is most similar to the gonad-inhibiting hormone of the freshwater prawn *M*. *rosenbergii* (AAL37948) [27] with an overall 70% amino acid identity, followed by the vitellogenesis-inhibiting hormone of *Rimicaris kairei* (GenBank #: ACS35348) [28] and the VIH of the lobster *H*. *americanus*. However, it is only moderately similar to the PeJ-SGP-IV (GenBank #P55847) of the shrimp *M*. *japonicus*. These findings suggest that there are homologues yet to be identified in *M*. *japonicus* (Fig 1).

### Sequence comparison of the 5 CHH family neuropeptides

To study the evolutionary relationship of these CHH family neuropeptides, sequence comparison was performed for the deduced CHH family neuropeptides (Table 3). The results indicated that the deduced mature peptides shared only moderate to low sequence identity (22%-66%) with each other. The deduced peptide for 4459–S and 4459-L shared the highest degree of amino acid identity (66.2%) because both transcripts shared exon one and exon two with identical amino acid sequences in the N-terminal end. Other sequences, however, shared only very low degrees of amino acid identity. For example, the deduced peptide of transcript 8108 shared 25–42% amino acid identity with other CHH sequences. The deduced peptide of transcript 28020 shared a higher sequence identity (22.3–54%) with other CHH family members. As a type II CHH neuropeptide, deduced peptide of transcript 14056 shared only 22.3–28.6% sequencing identity with the rest of the CHH subtype I sequences (Table 2). To further characterize these neuropeptides, pair-wise comparison of individual sequences was performed with selected shrimp sequences that showed the highest sequence homology in the original BLASTX and BLASTP analysis (Fig 1). When the deduced peptide of transcript 4459-L was compared with LvCHH, they showed 100% sequence identity in both the signal peptide and mature peptide regions. For the 4459-S form, it showed 97.2% amino acid identity with LvITP (GenBank #EF156402) [15]. The signal peptide and pre-pro-peptide region of deduced peptide of transcript 28020 shared low sequence identity (25–32%), but the mature peptide shared high (86.3%) amino acid identity with PmSGPIII (AF104390) and PmSGPII (AF104388) of *P*. *monodon* [9]. High amino acid sequence identity was observed in both the signal peptide (87%) and mature peptide (91.7% identity) regions of transcript 8101 when it was compared with CHH of LvCHH (GenBank #KJ660843) [13] alone. Transcript 32710 shared moderate (i.e., 70.7–72%) amino acid sequence identity with FcCHH-I and FcCHH-II of *F*. *chinensis* [16] in both the signal peptide and mature peptide region.

**Table 3:** Amino acid sequence comparison of the deduced CHH-family member.

Transcript	4459-L	4459-S	8101	28020	32710	14056
4459-L	100	xxxx	xxxx	xxxx	xxxx	xxxx
4459-S	66.2	100	xxxx	xxxx	xxxx	xxxx
8101	39.2	41.9	100	xxxx	xxxx	xxxx
28020	54.1	44.6	39.1	100	xxxx	xxxx
32710	27.9	22.7	25.3	32.1	100	xxxx
14056	27.3	28.6	26.1	22.3	26.1	100

Numbers indicated the percentage of amino acid identity between different neuropeptide identified in this study.

Although the deduced peptide of transcript 14056 does not contain an obvious signal peptide cleavage site, an arbitrary location with the largest cutoff value (SignalP) was defined as the cleavage site using alignment data of 14056 with other type II CHH family neuropeptides. Since the gonad inhibiting hormone of the freshwater shrimp *M*. *rosenbergii* MrGIH (GenBank #AAL37948) [27] is the only top matched sequence and no CHH sequence from marine shrimp was returned as a top matched sequence in the BLAST homology search, we have used MrGIH sequence in the pairwise comparison. The deduced peptide of transcript 14056 shared only 68.8% amino acid identity with MrGIH. The most conserved region was located in AA21-32 and the N-terminal end of the mature peptide (Fig 1).

### Phylogenetic analysis of the spermatophore-derived CHH neuropeptides

To study the evolutionary relationship of these CHH family neuropeptides among themselves, phylogenetic trees were constructed separately for the subtype I and subtype II neuropeptides with MEGA 6.0 [22]. Since the locust ion transport protein (ITP) consists of sequences very similar to the CHH family neuropeptides, we included the insect ITP as a reference outgroup gene to study the phylogenetic relationship of the spermatophore CHH family neuropeptides.

**Fig 2:**
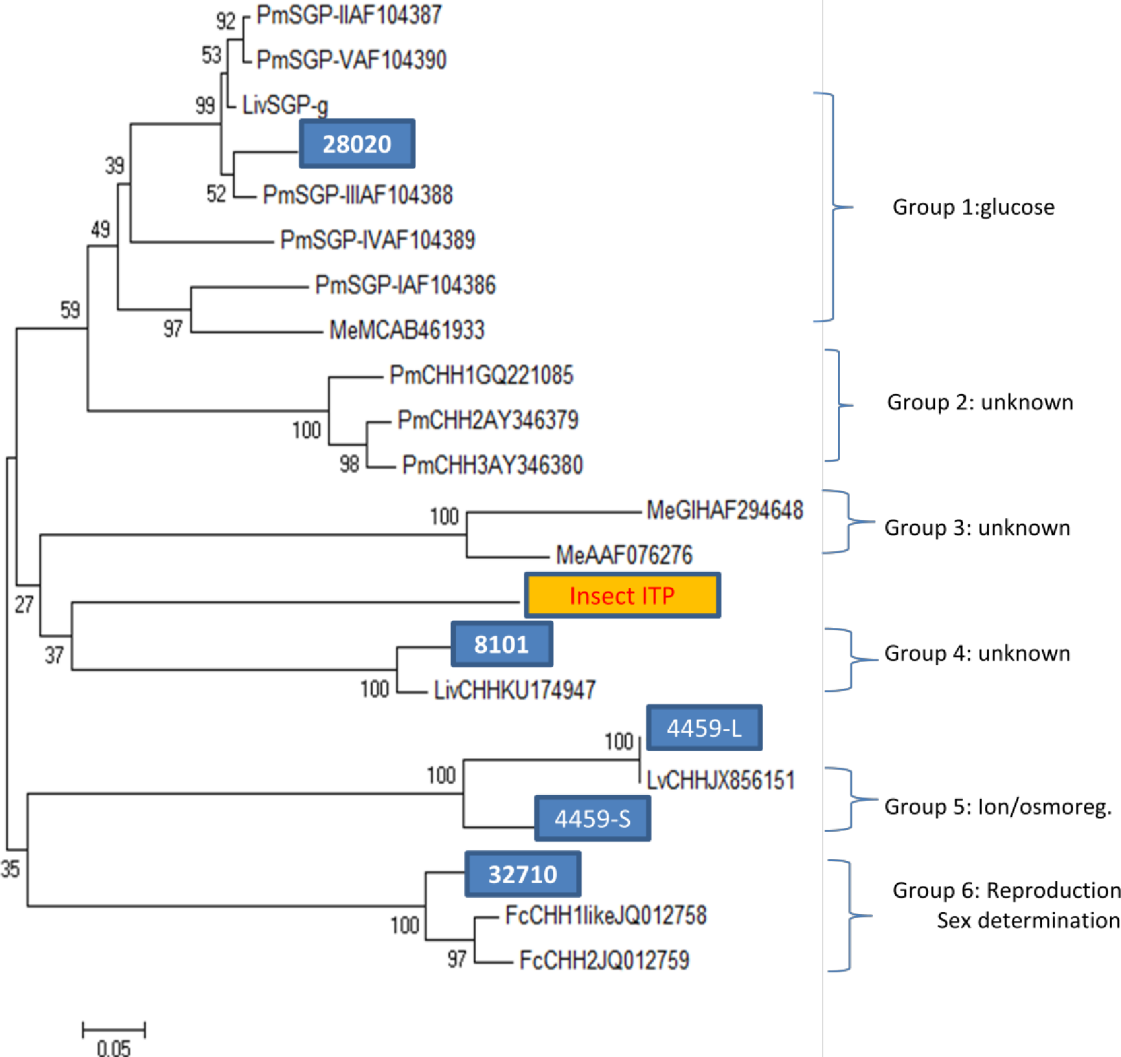
Phylogenetic analysis of spermatophore CHH subtype I transcripts of *F*. *merguiensis*. The spermatophore sequences and selected homologous sequences from other shrimps were analyzed by constructing a neighbor-joining phylogenetic tree using the program Mega 6.0. Transcript ID was highlighted and all the sequences can be divided into six major groups according to their reported functions.

**Fig 3:**
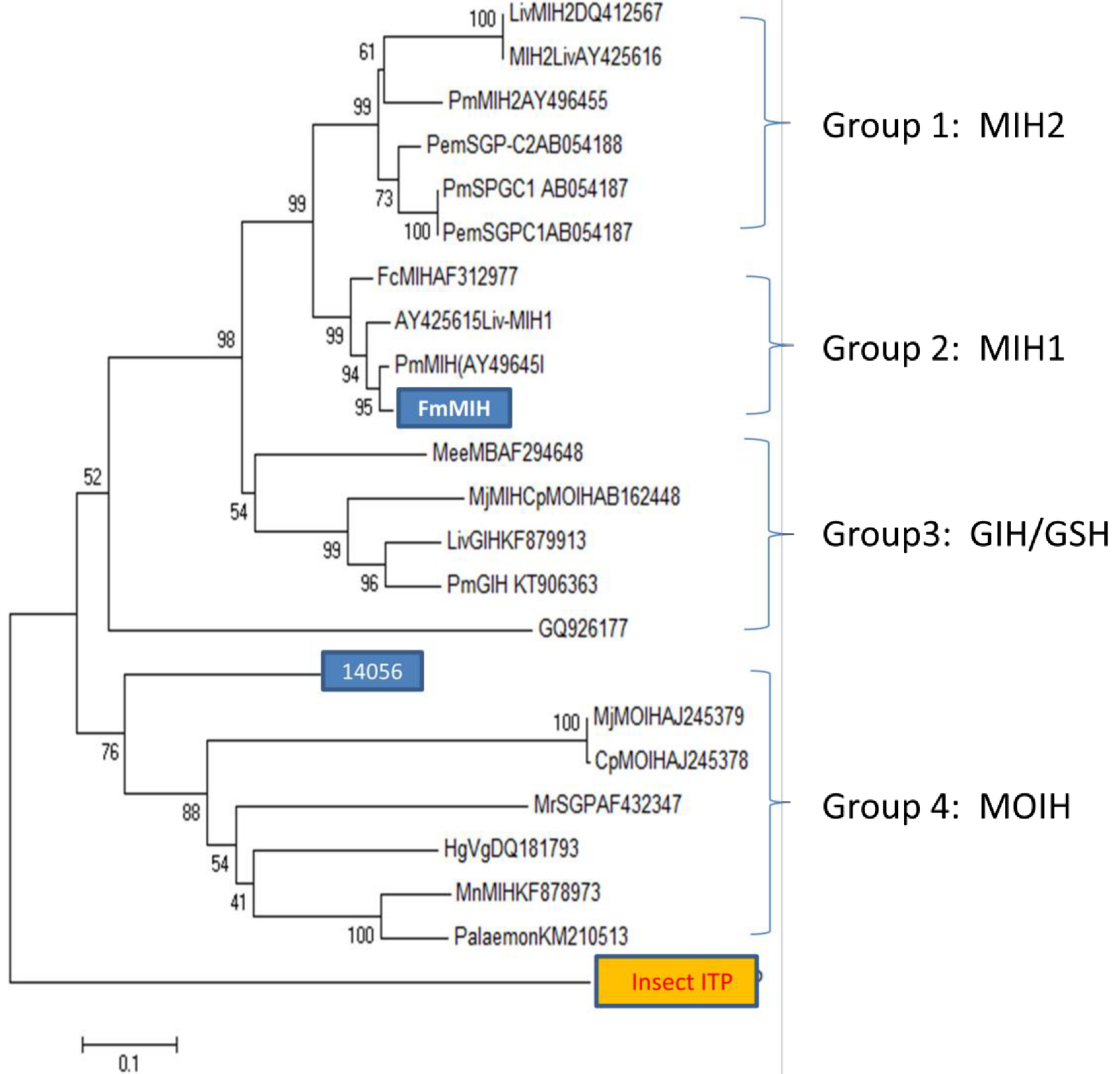
Phylogenetic analysis of spermatophore CHH subtype II transcripts. Transcript 14056 and selected type II neuropeptides of other shrimps were analyzed using the Mega 6.0. Transcript 14056 and a previously reported MIH gene (FmMIH) of *F*. *merguiensis* were highlighted.

To obtain an overview on the evolutionary relationship of *F*. *merguiensis* CHH neuropeptides with sequences from other shrimps, we have constructed a phylogenetic tree with all the top matched shrimp sequences compared with all the 4 subtype I spermatophore CHH family sequences (Fig 2). When the *F*. *merguiensis* CHH family neuropeptides and CHH sequences from other shrimps were included in the analysis, 6 distinctive groups of type I neuropeptides could be identified. Group I is the largest and consists of more than five CHH genes from *P*. *monodon* (PmSGP1-V) [9]. Deduced peptide of transcript 28020 is more closely related to PmSGP-III of *P*. *monodon*, sharing an overall 86% sequence identity in the mature peptide (Figs 1 and 2). LivSGP-g [23] and MeeCHH-B (#AB461933) are more closely related to PmSGP-II and PmSGP-III, respectively. Group two consists of sequences from *P*. *monodon* only (i.e., PmCHHi, PmCHH2, PmCHH3). However, none of the spermatophore neuropeptide sequences belonged to this group. Similarly, group three consisted of MeGIH and MeCHHA but no other sequence identified in the spermatophore belongs to this group. Group four consists of transcript 8101, LvCHH (#KU660843) and the locust ITP. The fifth group consists of transcript 4459-L, 4459-S, LvITP (#EF156402) and LivCHH (#JX856151). The genes for these neuropeptides were known to consist of four exons and three introns. They share a >98% amino acid sequence identity in both the signal peptide and mature peptide. Group 6 consists of transcript 32710 as well as FcCHH1 and FcCHH2 of the shrimp *F*. *chinensis*. FcCHH1 and FcCHH2 are subtype I neuropeptides that were also identified in the spermatophore.The subtype II phylogenetic tree results (Fig 3) revealed four different groups with different MIH or MIH like sequences from different shrimps. The deduced peptide of transcript 14056 showed low sequence similarity with group 1 (i.e.PmMIH2 (AY496455), group 2 (i.e. PmMIH1 AY496451) and group 3 (i.e. PmGIH; #KT906363) [9]. Instead, it is clustered with other non-penaeid sequences such as CpMOIH (3AJ245378) or MrSGPA (AF432347) of the group four in the phylogenetic tree (Fig 3).

### Expression studies of spermatophore CHH neuropeptides

**Fig 4:**
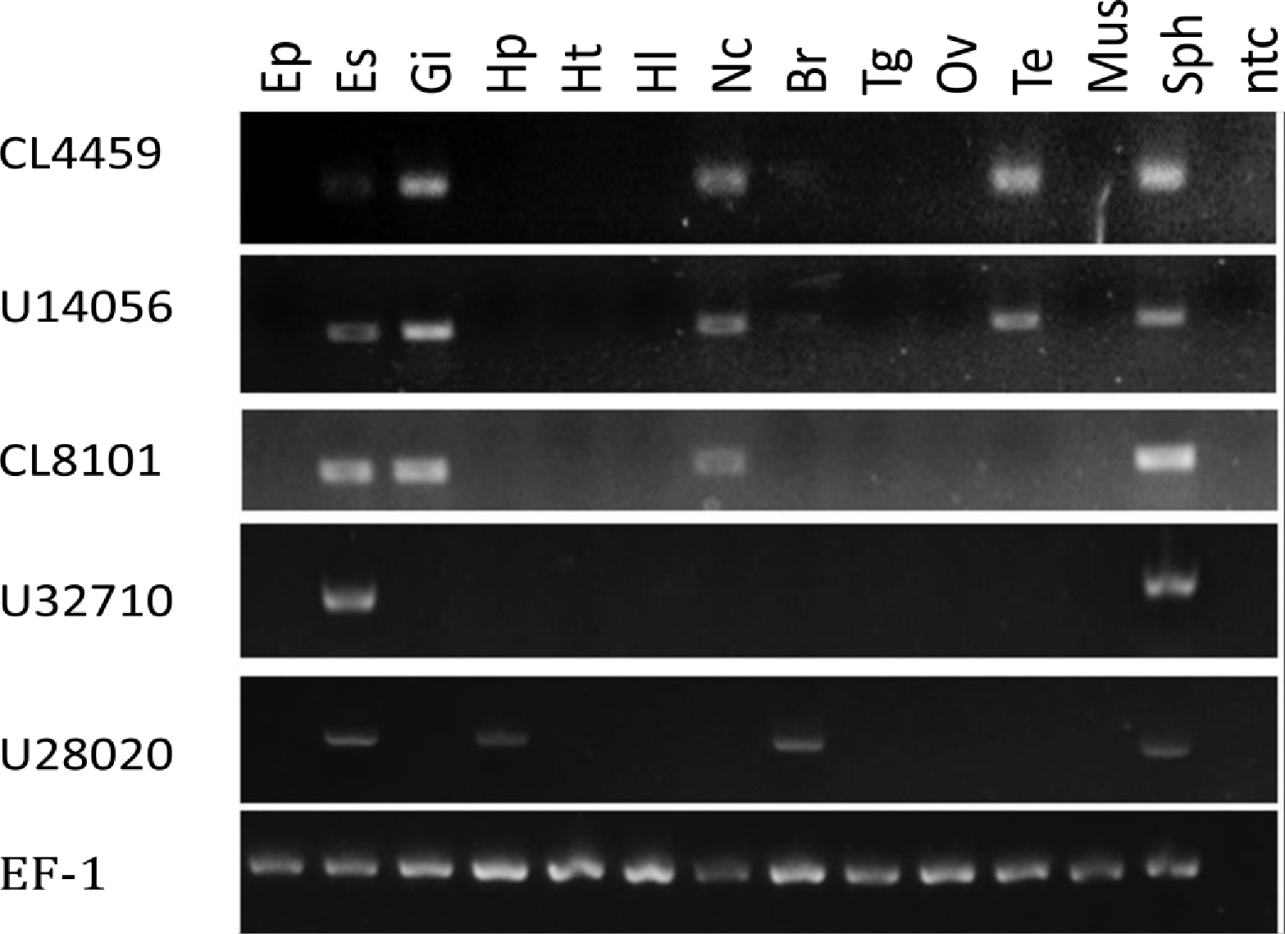
Tissue expression profile of the five CHH-family gene transcripts. Transcript ID was shown on the left and tissue include epidermis (Ep), eyestalk (Es), gill (Gi), mid-gut gland (Hp), heart (Ht), hemolymph (Hl), nerve cord (Nc), brain (Br), thoracic ganglion (Tg), ovary (Ov), testis (Te), muscle (Mus), spermatophore (Sph), no template control.

Previous studies also indicated that expression of these genes might vary depending on the life cycle, molt cycle or developmental stages [29, 30, 31]. In this study, RNA samples were prepared from adult male shrimp. As members of the eyestalk neuropeptide family, transcripts of these genes could be detected in the eyestalk in addition to the spermatophore (Fig 4). Transcript 4459 is the most widely expressed CHH family neuropeptide as it is expressed in the eyestalk, gill, nerve cord, testis and spermatophore. Other transcripts, such as transcript 28020, were also expressed in many tissues including the hepatopancreas and brain. Transcript 32710, however, could only be detected in the eyestalk and spermatophore. In addition, this transcript could be detected in the eyestalks of female shrimp as well.

#### Effect of eyestalk ablation on the expression of these neuropeptides in the spermatophore

**Fig 5:**
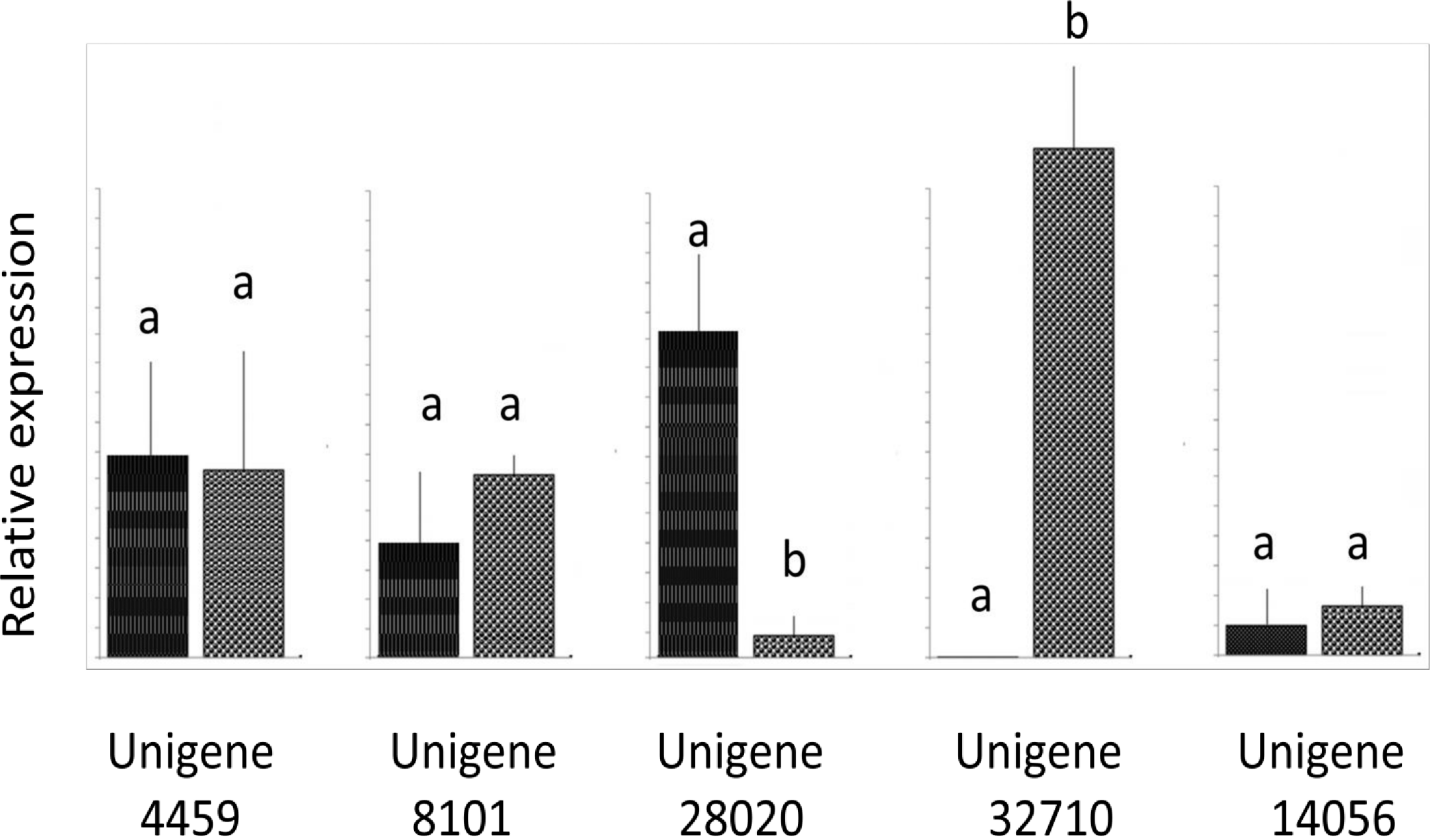
Expression of the five spermatophore CHH genes. Expression level of the gene in intact (left bar) and unilateral eyestalk ablated shrimp (right bar). Sample size is N = 6 for each group.Transcript ID was shown on the X-axis are for Unigenes 4459, 8101, 28020, 32710 and 14056. The relative expression level for each gene was shown on the Y-axis. Different letters above the standard error bar indicated significant difference (P<0.05) in expression level of each gene.

To study the potential regulation of these genes in the spermatophore by eyestalk factor(s) and to confirm the expression results from the transcriptomic dataset, we have performed quantitative real time PCR to determine the expression of these CHH-family members from the spermatophores of unilateral eyestalk ablated and intact shrimp (Fig 5). The results confirmed the changes in expression levels of these CHH-family neuropeptides in the spermatophore after eyestalk expression. Three different expression patterns could be observed in the unilateral eyestalk ablated shrimp. First, transcript 28020 appeared to exhibit a reduction in expression level after unilateral eyestalk ablation since transcript level of 28020 was high in intact shrimp but the level is virtually undetectable in unilateral eyestalk ablated shrimp. In contrast, the transcript 32710 level appear to increase after unilateral eyestalk ablation as the spermatophores of unilateral eyestalk ablated shrimp contain a much higher level of transcript 32710 than that of the intact group. Finally, transcripts level 4459, 8101 and 14056 appeared to remain unaffected by unilateral eyestalk removal as there is no significant difference of transcript level of these genes in both intact and unilateral eyestalk ablated shrimp (Fig 5)

## Discussion

Because shrimp are the most widely cultured crustaceans, much research efforts have been devoted to study the CHH neuropeptides in economically important species such as *L*. *vannamei*, *M*. *japonicus* and *P*. *monodon*. Therefore, many of the CHH family neuropeptides reported in the GenBank database are from shrimps. However, the annotation/naming of most of these CHH family neuropeptides is based on their sequence homology to the sequences of other shrimps or even different decapods. If the homologue of the new CHH family member in *F*. *merguiensis* has not been reported in other shrimps (as is the case for transcript 14056), it would be a mistake to name the newly discovered member according to the name of the top matched sequence. In a similar situation, although 2 type I neuropeptides were cloned from *F*. *chinensis*, they were named FcCHH-like despite their low sequence identity to CHH of other shrimps [16]. Therefore, to avoid the complication of different annotation/naming based on incomplete information from other crustaceans, a specific name for these neuropeptides in *F*. *merguiensis* was not given in this study. Additionally, in this study, if possible, only shrimp CHH neuropeptide sequences were selected for the pair-wise alignment analysis to study the possible evolutionary relationships of the shrimp sequences and how these sequences evolved among the shrimps. In this regard, transcript 4459 and *L*. *vannamei* CHH are considered homologues as they share >98% amino acid identity. However, transcript 32710 is also considered a homologue for FcCHH. Although they share only 70% amino acid identity, they share major similarities in specific region in the alignment (Fig 1) and cluster together in the same group in the phylogenetic tree. Both transcripts can be identified from the spermatophore. Unlike the MIH or other subtype II sequences, which were evolved later as compared to the CHHs [11], transcript 32710 and FcCHH evolved at a much earlier time in the evolutionary history of the CHHs. Sequences for transcript 32710 and FcCHH were more diversified as compared to other CHHs. Therefore, even though they shared only 70% aa sequence identity, we would consider them homologous. Therefore it would be interesting to identify important region/domains in both sequences that distinguish them from the other CHH molecules. In summary, it is expected that homologues of transcript 32710 or FcCHH-like [16] in other shrimps such as *P*. *monodon and M*. *japonicus* are yet to be identified.

Although the eyestalk CHH/MIH/GIH family neuropeptides were initially cloned from the eyestalk, subsequent studies have confirmed that some members are expressed in other neuronal tissues such as the brain and thoracic ganglion. Furthermore, in many recent studies, increasing evidence indicates that CHH family members are expressed in other non-neuronal tissues. Here, at least five CHH family neuropeptides can be isolated from both the spermatophores of intact shrimp andunilateral eyestalk-ablated males. By RT-PCR, we demonstrated that most of these transcripts were expressed at high levels in the eyestalk. Moreover, most of these genes were also expressed in other tissues (Fig 4).

Transcript 4459 could be detected in the gill, nerve cord, testis and spermatophore with notably high levels as compared to the eyestalk. Unlike other CHH-family transcripts, the gene for transcript 4459 most likely consists of four exons and three introns. In the spermatophore, the four-exon transcript is present and there is no transcript derived from exons one, two and four. In other tissues, such as the eyestalk, both transcripts co-exist at certain stages of the molt cycle. In general, most of the crustacean CHH-family genes consist of three exons interrupted by two introns. Only a few subtype I CHH genes are known to be composed of four exons and three introns. The four-exon CHH gene may undergo alternative splicing process and resulted in the generation of either a long or a short splicing variant. The presence of a four-exon and three-intron CHH gene was first reported in crab [32]. For example, in the crab *Carcinus maenas*, the pericardial organ CHH is a long form splicing variant generated from exons one, two, three and four. The final splicing product is a four-exon transcript with a silent un-translated exon-four. The sinus gland CHH (sgCHH) is the short form splicing variant generated from exons one, two and four. In the shrimp *L*. *vannamei*, the short form CHH is generated from splicing of exon-three and the final splicing variant is produced from exons one, two and four. The long form CHH is generated from transcript containing sequence of exons one, two, three and four. This splicing variant is expressed mainly in the gill Presence of the stop codon in exon three produces the ion-transport protein like (LvITP) with sequence divergent from the CHH of most other crustacean CHH. In this study, four subtype I CHH-like members were identified in the spermatophore. Only transcript 4459 carry coding sequence derived from four exons. In the spermatophore, the 4-exon transcript is present and there is no transcript derived from exons one, two and four. In other tissues, such as the eyestalk, both transcripts co-exist at certain stages of the molt cycle. In all these CHH-like subtype I genes, the first intron interrupted the signal peptide region and the second exon ended in the codon for the 40th amino acid (aa) of the mature peptide. In the crab *Carcinus maenas*, an alternative splicing event occurred in a CHH-like gene that produced two different cDNAs with identical deduced sequences in the N-terminal end. In *F*. *merguiensis*, since the two transcripts produced by the same gene differ mainly in the C-terminal end, these two proteins (i.e. the long and short form) should have a different function(s) in the shrimp.

### Expansion of the crustacean CHH family neuropeptide superfamily

At present, there is no definite number of CHH family members in shrimp. Results from molecular studies and genomic DNA library screening have provided evidence that the shrimp genomes contain multiple CHH genes and most of these genes are arranged in a cluster [33]. It has been estimated that the shrimp contain at least >20 different CHH neuropeptides [11, 33]. The total number of type I genes is much larger than the total subtype II members of the same species [11]. The success in matching or identifying a homologue from one species but not the other indicates that the same gene is most likely present in other shrimps. For example, transcript 8101 is homologous to *L*. *vannamei* CHH-like (GenBank #KU174947), but a similar gene in *P*. *monodon* has not yet been reported. Therefore, a homologue for transcript 8101 is most likely present in *P*. *monodon*.

On the other hand, transcript 28020 shared a high degree of sequence identity and clustered with PmSGP-II (#AF104387), PmSGPIII (#AF104388) and PmSGPV (#AF104390) in the phylogenetic tree, and we should therefore expect to identify similar numbers of homologues in *F*. *merguiensis*. If we were to extrapolate this finding to estimate other type I genes in the phylogenetic tree, we estimated that a single shrimp contains aat least 10–12 different type I genes.

The neuropeptide encoded by transcript 14056 represents a novel CHH-family member expressed in the spermatophore. The presence of glycine residue located at position 12 placed it as a subtype II CHH-family member. The BLASTX (and BLASP) search results indicated that the top best match shrimp sequence is Liv-MIH. However, the overall amino acid sequence identity in the mature peptide between the 2 sequences is <61%. Furthermore, when we compared the translated product of transcript 14056 with MIH-I and MIH-II sequences of *P*. *monodon*, the results showed that the deduced peptide of 14056 shared only 58% and 61% sequence identity to the PmMIH1 and PmMIH2 respectively. Further analysis of transcript 14056 by SignalP indicated that it did not appear to have a signal peptide sequence. In contrast, all the LivMIH, PmMIH are predicted to have a signal peptide. Therefore, the translated product of transcript 14056 is unlikely a secreted protein. In the BLASTX search results mentioned from the above, the top matched BLATX search sequence for transcript 14056 is the GIH of the fresh water shrimp *M*. *rosenbergii*. Transcript 14056 show a much lower degree of amino acid sequence identity to the GIH of *L*. *vannamei* and *P*. *monodon* (i.e. 55–57%). To interpret the results, we are aware that the functions of MrGIH have not been tested. Furthermore, transcript 14056 could be detected in the eyestalk, gill, and hepatopancreas of juvenile shrimp before maturity is reached. Therefore, it would be premature to name transcript 14056 as FmGIH of *F*. *mereguiensis*. To summarize, it is most likely that the translated product of transcript 14056 would not have a major functions in both molting and reproduction.

#### Phylogenetic implications from the study of spermatophore CHHs

Although there are more than 100 crustacean CHH family neuropeptides reported to date, phylogenetic study results provide little information on the total CHH family members identified in other decapods such as crab and lobster because most of the cloning of CHH family members is incomplete for each species. There are far fewer CHH-related sequences reported in other decapods such as crab, crayfish and lobster. In this regard, we have used only CHH family neuropeptides from several most studied shrimp species as a reference for the analysis of CHH neuropeptides in *F*. *merguiensis*. At present, *L*. *vannamei*, *M*. *japonicus* and *P*. *monodon* are the major marine shrimp that have the most CHH family neuropeptides reported in the public database.

#### Functional implications from the phylogenetic study of spermatophore CHH neuropeptides

The low amino acid sequence identity among the spermatophore CHHs suggests that they may have different functions in shrimp. The results from the phylogenetic tree analysis indicate that other similar sequences must exist in the penaeidae.

Biochemical characterization of CHH family neuropeptides began in the 1980s using chromatographic techniques, and CHH family members were restricted to CHH, MIH and GIH [34, 35, 36]. The hyperglycemic functions of the CHH have been reported in many decapods. It is also the most abundant CHH family neuropeptide expressed in the eyestalk. This finding may be explained by the existence of large numbers of subtype I group I genes (Fig 3). In *F*. *merguiensis*, transcript 28020 may be considered a CHH having a glucose metabolic function. Transcript 8101 and transcript 4459 are clustered in different branches of the phylogenetic tree suggesting that they may have different functions. Since FcCHH-like and transcript 32710 can be detected in the spermatophore, they may also share similar reproductive or sex determination functions (Fig 3).

The mandibular organ inhibiting hormone (MOIH) is the most recently discovered subtype II CHH-family member. At present, the presence of a MOIH was only reported in the crab *Cancer pagurus* [37, 38]. Its presence in the shrimp has not been verified. Results from phylogenetic tree analysis also suggests that transcript 14056 is related to the MOIH of the crab [37]. It would be interesting to determine whether this transcript has MOIH activity if we can locate a homologous mandibular organ in shrimp.

## Conclusions

In conclusion, five eyestalk CHH family neuropeptide members were identified in the spermatophore of *F*. *merguiensis*. Using molecular and phylogenetic approaches, we have provided evidence that these neuropeptides may have different functions in the male. At present, the list of the CHH family neuropeptides has not been completed for a single shrimp species, and the potential in “wrong labeling” of CHH family neuropeptides in crustaceans cannot be ignored as most of these neuropeptides share relatively low sequence identity. Furthermore, we may have to re-evaluate the previously accepted generalized function, cross-bioactivity as well as the multiple (pleiotropic) functions of this large group of eyestalk neuropeptides and perform research on their specific functions.

## Supporting information

S1 Fig Nucleotide sequence for transcripts 4459, 28020, 32710, 8101 and 14056. (DOCX)

S2 Fig RT-PCR detection of transcripts 4459L and 4459-S in the eyestalk (Es), gill (Gil) and spermatophore (Sph) of the *F*. *merguienesis*. (DOCX)

S1 Table Primers used for qRT-PCR expression studies of the five spermatophore genes. (DOCX)
