# Supplementary material for: Functional and evolutionary implications from the molecular characterization of five spermatophore CHH/MIH/GIH genes in the shrimp Fenneropenaeus merguiensis
Source: PLoS One. 2018 Mar 19;13(3):e0193375. doi: 10.1371/journal.pone.0193375 (PMC5858750; doi:10.1371/journal.pone.0193375)
Supplement: S1 Fig — (DOCX) [file pone.0193375.s001.docx]

| 4459rtF | 5’-TCCTCGTCGTCTTCGTCAGG-3’ amplicon size 157bp |
| --- | --- |
| 4459rtR | 5’-GGTTGTAGCAGTCCTCGCAG-3’ |

Transcript 4459

TTCGGCGAAGCATCCCCGTTAACAGTAGAGCCAAAGCCCTTGTTCGGTCTCTCTCGCTCGCGCTCTTGCTCTCAGACCAGCATCCCGAGTGAGAACGCAATTACAGGTGTACTACCTTGTGTGTTATCGTTGCAATATCCACCTGCATTTGCAACCGCAACCATGGTCAGCTTCCTCTCACTTCGCATGGTGTGCTCCGCCGCCCTGGTGTCGCTGCTGGTGCTGGCCCTGTCGTCCCGCAGCGCCTTCGCCCGCTCCGTCGACGGCGTGGGGCGCCTTGAGAAGCTGCTGTCGTCCTCGTCGTCTTCGTCAGGCTCTTCTTCCCCCCTGGATGCTCTCGGCGGCGACCACAGCGTGAACAAGCGCGACACCTTCGACCACTCCTGCAAGGGCATCTACGACCGGGAGCTCTTCAGAAAGCTGGACCGCGTCTGCGAGGACTGCTACAACCTGTACCGCAAGCCCTACGTGGCCACCGAGTGCAAGTCCAATTGCTTCGTGAATAAGAGGTTCAATGTCTGTGTGGCTGATCTCAGACATGATGTCAGCCGCTTTCTGAAAATGGCTAAATTTCTGCGCTATCCCTAATGGTTGAAGGCTATGGAGTAACTGCTACGCCAACTTCGTATTCAAGCAGTGCCTCGACGATCTCCTTATGGTCGACGCCATTGACGAGTACGTGAACACCGTCCAGCTGGTAGGGAAGTAAAGGCAGAAGTCTCTCAGGACGCTAATGTGGAGGAAACAAGAAAAAAAACAGGAAAAATATGGAAATATTCACGACAACTAGAATCATTTTTGAAAGCCCCTTGTCTGGAGACTTGGGCAAAATGGGGCCAAAGAAAATAGAGATGCAGTAAAGAATTATTTTAAGTTGTCTTCTTCAGGAAAACATGACGCTCTATGGCAGCTCCTTTATCATTCCATACTAATTAGTAAAATATGATTTCTACCTAGTTATGAAATACATAGTAACTATATTATGCTAAGTATTTATTGAATATCATATATTTATTTTCATAACCTTTTGAAGTGGCATTTGAACGATTCTCACCACTGTCCTTCTCCCATTCATTTCTTCCTTCTCCCTCTCCTCAACTATTTATGCAATTCTGGTTATACGCTTTGAAAAGTTATTTTTGTATGGTCGATAATCATTAATCATAATACAGTGTACAATTCGGCATTTATGTGTGAAAGAAAGGCTAGGAAACCACACAGCACATGAAGGCACGTGAGATTCAGTATGGTTTCTTATCTGCGGCCTGTTGGGATCTGAAAATTATATTTTCTTGAATCTGTAGACCCCCTTCGCACAAAATGAGGTAAAGTTACAAAGTTCTGAGCTGGAAGAAAAATCCAGTGCTACTTTGTCTTTCCATTCCCCTACCCTTTCCCCCACACAGAGGGGGAGGGGACACAGCGATTTGCTTGCTAACTTCCACAAAATCAGATCTTTCCCCTTTGAAAATTCTTGTTGTTTCAGTCGGTTGAGTCTTTTAGAAATAATATCTTCCCCAACTCGAAGCAAGGTATTCTTCAGCTTCTTAAGTAAAAAGAGGAAAGGCACGATCACCTGCTTTTCCAAATCAAGAGAAAGAGAATGCGAAAGAGAGAAATTCTTGATCTCTGTACTTACCACTTGTTGCTGGTGGTGCGTGTGGTGCCCGAACCCTAGTTATGTAACCAAAGATTAGAATTTAGTGGTATCCTTAACTTTTGCGTTTCTGTTGGATCCTTGAGATTTTGAATAAATTATTATAATCAAAAA

| 8101rtF: | 5’- GGATGAGCGGAAAGTGGTTC -3’ amplicon 153 bp |
| --- | --- |
| 8101rtR: | 5’- CTTCTCGTTTCTTGCCGCTT -3’ |

Transcript 8101

TTCAAACGTTCCGGTGGTAACAAGGAGCTCCTCGACAAGCCTTAGAGCGGGAACTCGTGTATAAACTGTAGCTAACTTACGAAGAACACAGCCATGGACAACAAGATCGCCTTCGTCTCTGCATCAGTTCTCCTTCTGGTCGCCGTCCTGGCATCGCACAACGGCGTCCATGCGAGGTCCGTCGTCCCCGAAGGCCTCCAGGAACTCGAAATTCCTCGTCAGGAGAGCGACATGTTCGCCGTCAGAAGAAAGAGGCAGGTCTTCGACGCCTCGTGCAAAGGGGTGTACGACAGGGGCCTCTGGGCCAAGCTCAATAACGCGTGCTTGGACTGCCAGAACATCTATAGGGGGAATCCGGCCATTGAGGGGGAATGCAGGCAAAACTGCTTCGGTACAGAGGTCTTCTACGGATGCATCAAGGCCCTAAAACTACCCACGAAAAATTACTTATACTTTGCCGAAGTCTTGAGAGAAAGCTAGTGTGTGGGAACAATTTATAACTCAAGAATTAGTCATGAGAAATCGTTGCAGCTGAAAAAAAAAATATATATATCTTACACTCGCAATCCCTCCATAAGCGGTTATACTCTACCGGATTGCCAAGTCCCTCGTTAAAGAAACCAATTCCTTGCTGAATTATTTTTCCAGCGCAGCAACAACAACAAAAGAATTTCTGTTTGTCTCGCGCAGTGATGTGGAGTGTCGGGTGGTCCATTGGAGTGACGTGGATCAGTTTCTTGGAGTATTGGGGTTTAACTGAAAGGTCTTTTAGAGAAATTACGTAAGATTGGAAGGATGGGAAAATCTGCAGGAGACTGTGGTGGTTGGAGAAATTGCACACGAGTAGGATGAGCGGAAAGTGGTTCTGGAAAGGAAAGTGGAAGATGGTGTAAATATAGAAAGTGAAGACATATTGGAGAAAGGAAGAAGAGAAATATAAGCGACAAGAAACGAGAGAACGGAAGAAGAGAGATATAAGCGGCAAGAAACGAGAAGAAGGAAGAGTAAAAGACCAGGAAGGAGAAGTATATAGAATAGACCCCAAGAAACGAGAAGAGAGGAAAGTGGGAAAAAACGCAGAATACAGAAAAAATGAAGATGGATAAATGGAAACAAAAACCCCGAAGTGAATCATTCTCTTCATTTATTCGGAAGTGAAGAAAAGAAGAGACGTGACAGTGAACACGTGAACTATAAGTATTATCGGCCATTATTTACCATATCCTGTATCCTCATTGGGCATTCCTCGAAATAGGTTAGGAAGACCGAAATTGCTGACGTCATCCACGGCAGTTGGCAAAGACCTTTTTTTAAAATTCGCGATAAGATTATAGTTTCACTTCGTCTTTTTTCATGAATTGCATTTAATACGTACATACATACATGCATGTGTGTATATATGTGTACATATATGTATACATGCATGTATATTTTTCATGTGTTGTTCTAGTCTGATATGATTATAATTGTTTCATATTTTTGTACCTGCTTTACATATTTAAATAATTTATGAATTAGA

| 28020rtF: | 5’- TTCAGCAAGCGAGCGAACTT -3’amplicon 107 |
| --- | --- |
| 28020rtR: | 5’- TCGCGAAACACGTTGTAGCA -3’ |

Transcript 28020

GGGAGATTTGCCTTCGTCAGGAGAGCCACAGCCGCCAGAAGGACCGTTTCTGTTCACAGTTTGACTGCCTACATTATCCTTTGCTATGATCGCCCTTCGTTTGATGGCCGTGACCCTGGTGGTGGCGATGGCGGCGTCGACGACCTGGGCTCGCAGCTTCAGCAAGCGAGCGAACTTCGACCCTTCCTGCGCGGGCGTGTACGACCGGGAGCTCCTAGGAGGGCTGAGCCGCCTCTGCGACGACTGCTACAACGTGTTTCGCGAGCCCAAGGTGGCCACAGAGTGCAGGAGCAACTGCTTCTACAACCCGGTGTTCGTCCAGTGCCTGGAGTACCTGATTCCGGCCGACCTGCATGAGGAGTACCAGGCCCACGTGCAGACGGTGGGCAAGTAGGGCCGCTCGACCTGCCTCGACCTGCTTCCCGAACGCACGCCAACGGCCAGAAGACGCAGAGGGGATTTTGGGATTTGTTTAGGGGTAACTGGCGCATTTTAACGATCCTGTACGGATTTTGATATGACTTTTCATATACTAGATTGTGATGAATCTCTTAATGAAGTATGTTGGAATTATGCCTGCTGGCGGCGCTGGCTGCCTCGACGACCTGGGCTCGCAGCTTCAACAAGCGAGCAAACTTCGACCCTTCCTGCAGGGGCGTCTACGACCGGGAGCTCCTGGGGAAGCTGAACCGCCTGTGCGACGACTGCGACAACGTGTATCGCGAGCCCACGGTGGCCACGGAGTGCAGGTGGGGCTCCTTGCAGATCACCCAGATGTGTGTAGGATATCTCACATATTCCCTATTTTTTATCTCAAATAGTCCCCCAGTCTCTTGTATTCCCACACCTTTACGCATTAGCAATACATACTGAGTGACTGTCCTCCATTGCAAGCATTTTAGGGTACTGCTTACCTAGTGTCTAATAAGCTATCACCCTCTGGCTTTCCGAAAAATGACTTGTACTGATAATTGTTATCGTGTCCAGCACATGAATAATTCTACACCCTAATTAATATACAACTATTTTCGGGGCATAAATCACAGGATATCTCACGTATTCCCTCGGTTTTGTCATCTCAAAGAGTCCCTCAATCTTATATATTCCCACATTTTTTACGCATTAGCAGATCATACTGGCCTTTATACCGAGCGACCACTTCACTGCAAGTATGTTAAGGTACTGCTTACCTAATGTCAAAGAAGCCACCAACTTCTGGTTTTCCGAAAAAAATAAATAAATAAATAGAGTCGTATAAATGATTGCTTTCCTGACCAACACTTGGATAATTCTACTTCCTAATTGAACTACAACTGCGTCGTTTGCCGAATGGTCTCCCCGTCCAGGAGCAGCTGCTTCTACAACCCGGTGTTCGTCCAGTGCTTGAAGTACCTGATTCCGGCCGACCTGCACGAGGAGTACCAAGGCCATGTGCAGACGGTGGGCAAGTAGAGCTCGACCTGCCACGACCTCGCCTCGCGATCTCACGCCGACGGCCAGAAAAATGTCAGGATTTGTATCGGGCTGACAGGCGCGTCTCAAAACTCAACGGATTTTTGTACGAACTTTCACACACCAGATGAATCTCCAAATGAAGTGTGCTGGAATTTTTGAAATATTATTTTCCCACTTTTCATAACAAGTAAGACAAACATTAAAGACAAAAAGGGAAAAGGGAGGTAGAAGTGTG

| 32710rtF1: | 5'- ATCGGCCCACAGACACTACA-3 amplicon 171bp |
| --- | --- |
| 32710rtR1: | 5’-TCGAGGCTCTCTTGCCTCTC -3 |

Transcript 32710:

TCCAGATCGGCCCACAGACACTACAATCCGAACCTAGTGGCTTCCTTCGTACTACTCACTATGATCGCCTTTCGGATGATGACTTTAGCGGTGGTCGGAGCGTTGTTGATGACTCAGGCTGATGCCCGCAGTCGTTTTCTCCTCATGCCTCTCTCCGAGAGGCAAGAGAGCCTCGAAGAACGTGATATCTATCCCTCGGAGTGCCAAGGATCGTTCAACCTGGCTGCCTTGAGAGCGGTGAATGATCTGTGTCATCAATGCGGAAACGTAACTCGGGATCATAATACCGAAGTCAATTGCAAGGCAAGCTGCTTCAACAACACTATCTTCGCCGGTTGTCTCGAGTTGCTGCAACTGCCAGAAAGTCAGAAAACTACGTACACAGATCATGTGGCGTTGCTGGGTAAGTAGTCCCACTCCGCCTGCAGTGACCACACGCTGCTGCAAGATATCGGAGACAGGGCGTTCAGGGACTAACTGTTTGTCACGAACGAAGGAATGAACTGTGTTTCCTCACGCCTGAAATAAATG

| 14056rtF: | 5’-CGACTGATGGCATCTCCACG-3’ amplicon 160 bp |
| --- | --- |
| 14056rtR: | 5’-CGTAGATGTTCCGGTTGCCC-3’ |

Transcript 14056

AAGGCACCACGCAACGAGAGTGACCCCCTGCTATCCCCCGAAGGGCAACTAACTTCTTCCAGGCGCCGCCTCCTGGACCCTCGCGCACTCTCTGGCCCCTCCCACACCACTGCCGCCGTGTCCTTGTCCTTCGCCACTTCAGTAGGGTCGGCATAACCCTTCCTGGCGCACGTCCCAGCACATTGCGAGACGGTCGAGCAGCGGTCCTTCGAGCGAGGCATGTGGGCAGTGCCCTCCAGTAGGCAGGGAAGCGGACATTTCTCTCCCAAGGCGACCCGACTGATGGCATCTCCACGTAGAACCCCCAGCATCCTAAAGAAGGCGTGCCAGGTGGCGTTGGTGGCAGCCGTGTTGTACGGCCTCCTGACCGCGCCCGCCTCCGCCAGGTTCATCGACGACGAGTGCGTGGGCGCGATGGGCAACCGGAACATCTACGAGAAGGTGGCGCGCGTCTGCGACGACTGCAGCAACATCTTCCGCCTTCCGAACGTGGGCGAGAGTTGTCGGAGAAACTGCTTCTACAACGAGGACTTCCTGTGGTGCATCATGGCCTCGGAGCGGCACGCGGAGGTGGAGCAGTTCAACAGGTGGATCAGTATCCTCAAAGCGGGTCGAAAATAAGACGCACGTCAAGCCCATCCCTGCCGCCTGTCCTTCAGCTTAATACTACGAGTCTCCTCTCCTCCTGCTGCTCCTTCGCCTTCTCCCGCGCCCTACCCTTCGCCGCCGCCACTCACCAAGTAGGACAGGCGACGCCCACGCAGACATTCGTCAGCCGACCGCCGGATGCTGTTATTGTTTCGAGTGTGATCGATGGCAACCCCGTCCAGCGCCGTCGAGACAAACGTTTGGAGTTCCTGAGCAGACGTTTCTCTAAGCTGATCTTGATTCGATTCGTTTAGGTTCAGTTTCCTTTGTTTTCTCAAGGGACCACTACTAAAGCTAATCATTATTTATCACTAAACGCAACTTTCACGGACACCTTTACTCTCGCTTTCCCATTACCCTCTCTCTTTGTTTTTTTTTTTATCTCCGTCTCCATGTCAAGGAATCTATTTATCTGAATTCACTTTCCGATCCCCCCCCCCAAACCTGCAACTAAATGCAACTTTATATCACAGGCACTAATTATGGATCTCATATACATTACCTCATCTGCTTTCTGGCATTTTTTTCTTTTCTTTTTTACTTACTTGGATTTGACTAGAATATATTTTTACATATATTTATATATTTTTGTATTTAAGATATTTATTTATTATTCTTAATTTTAAACGAAGCTGATGTGTCACTTATATCGAATATTTCTCTCTAACCCGCTCTGGGAAAATGTGTCTCATGAGGCTGGCCCGAATTCACTGAAGAAGAGGATCAACATTCTATTATCAGAGCGCAAATACAAGATTATTACTGTATCGCTCTGCATTCTATTGGCTCTGCATATGGTTTTACTAGCTTTTTTACCAAATGGAGCAATATTTGTAAGCATATATATATACAAATATAAATATATATACATATATGAATATACAGTACTGTATACAGCTAAGTCATCCAGTGTAAAATGCGTCTGAAAGGAGTGAAGAGAGATCCTTGAAGGTAAAAGTTATGTATAGTTATGAGCTTTCAGAAACATGGTCACATCTTTATTTCAGTGTCCCGTATCTACTTTGTCAATATTTGGAGTCATGCGTGTACCTTGTGTCTCATCATCCAATACTCACCACCGGGGCTTTGGGTAGGCGGGTGCAAGTTGTCTAGCT

EF-FP: TGTTCCAGCGAGACAAGCCC 252 bp

EF-RP:

Transcript 1036

GCAGACGACGCTAACATGGCAGCGTGTCGGGTGCTGCGGTCGCTCTCCTCCTCCCATTTATCACCTTGTTTTAGGGCCTACCAGTACCACCATGGAAGTCTTGCCAGTCTAGCGAGGTCCAGGGGCTTCCCGGCACTCTTCCAGGTGCGCAATTATGCAGAGAAGAAGGTGTTCCAGCGAGACAAGCCCCACTGCAACATTGGTACCATTGGTCATGTTGACCACGGCAAGACAACGCTGACGGCTGCCATCACCAAAGTGCTGTCTGACAAGGCCCTTGCAAATGCAAAGCGGTACGACGAGATTGACAATGCCCCCGAGGAGAAGGCCAGAGGTATCACCATCAACGTTGCTCATATTGAGTATGCGACAGACAAGAGGCATTATGGCCACACTGATTGCCCAGGCCATGCCGATTACATTAAGAACATGATCACCGGCACTTCACAGATGGACGGAGCCATCCTGGTGGTGGCGGCGACAGACGGTGTCATGCCGCAGACCAGGGAGCATCTTACTCTTGCCAAACAGATTGGCATTCAACACATTGTCATCTTCATCAACAAGGTGGATGCTGCTGACGAAGAAATGATAGAGCTAGTGGAGATGGAGATCCGTGAATTGATGACGGAGATGGGCTACGATGGAGATAACATTCCCGTTGTGAAGGGCTCTGCTCTCTGTGCCCTGGAGGAGAAGAACGACGATATTGGAGTGAAGGCTATCGAAGAACTCATGAGTCATGTTGATGAGGAGATTCCAACTCCTGTGAGGGAATTGGACAAACCATTCTGCCTCTCTGTGGAAGGAGTTTATTCTATAGCAGGACGCGGCACAGTGGTGACAGGCCGGCTGGAACGTGGCAAGATCAAGAAGGGCATGGACTGTGAATTCATCGGCTACAACAAGAAGATCAAGAGCACCATCACGGGCATTGAGATGTTCCACCAGATCTTGGAGGAGGCCCAGGCGGGGGATCAGCTGGGGGCCCTGGTGCGCGGCCTCAAGAGGGACGACGTCAAGCGCGGCATGGTGATGGCCAAGCCGGGAACAATGAAGTCGCATGACAACGTGGAGACACAGGTGTACATCCTCAGCAAGGAGGAGGGTGGTCGCAGCAAGCCCTTCACCTCGTACATCCAGTTGCAGCTCTTCAGCAAGACCTGGGACTGTGCGGCCCAGGTCATTGTCACTGAGAAGGAGATGGTCATGCCAGGAGAAGATGCCAAGCTGACCTTGCGACTGCAGAAACCCATGGTCATGGAAAAGGGCCAACGCTTCACACTGCGTGATGGCTCAGTCACCCTGGGAACGGGCGTAGTCACCAACATCCTGAACAACCTGAGCGAGGTCGAACGCTCCGACCTCATGGCAGGCAAGAAGGCCCGAGAGAAGAAGGCTGCTGCTGAAAGTAAATAACTTCAGGGAGAGAGGAAGTGAAAGAAGAATGATAGCTTGTGAATGCAGCAGATTTTTCACGAACACTGGGAGGAGTTGGGTAGAGGAAACCCATGAATGCTAAGGATATACAGGCTATAGTGTAACCTTGGGTAAAGCAATGCTGTTGGTGTTGAGGATATAATATATATATTTATTTTATTCCAAACCCTGTGAATGATAAAAGAAGAATGATCTTAGTGTCTTTTATTTTATTTGGACTTGACAAGAATCTTCATTGAATCAATGGGAAGTGGAATTTTGCATTATAGGTTGGAAATAAGTCTGAAAAATTACATTGTCGTAGAATTCACAAACATTAGAAAGTATGGCAGGTTGAGATAAGTATGTAAATATTAGAAAGAAAGAAATTTTGTTTCAAGTGAATTTGTCTGATAAAAAAAAAACAGCAAACACCACAGCCTATATTCAACTTGTAATTCAAACTGCAAAATTTAAAGAAATTTGTTAATGTGTACTGTATATAAACAAACCTTGAAACGTTAGTGCACTGTGAATCATGCACTGCAAATCTCTTCATGAAAATGTTAGGATTTAAATTCCTTTGGTTCATGACCAGTGATTATATGTATTGTTCAGTTTATTAACTTTTGTAAATAAAATTGAAATGTTAAAAAAAA

S1 Fig. Nucleotide sequence for transcript 4459, 28020, 32710, 8101 and 14056. Nucleotide highlight in green are location of primer design for RT-PCR detection of gene transcript for the spermatophore CHH family gene. The sequence of elongation factor 1 (EF1) is also included.
