# Supplementary material for: Functional and evolutionary implications from the molecular characterization of five spermatophore CHH/MIH/GIH genes in the shrimp Fenneropenaeus merguiensis
Source: PLoS One. 2018 Mar 19;13(3):e0193375. doi: 10.1371/journal.pone.0193375 (PMC5858750; doi:10.1371/journal.pone.0193375)
Supplement: S2 Fig — (DOCX) [file pone.0193375.s002.docx]

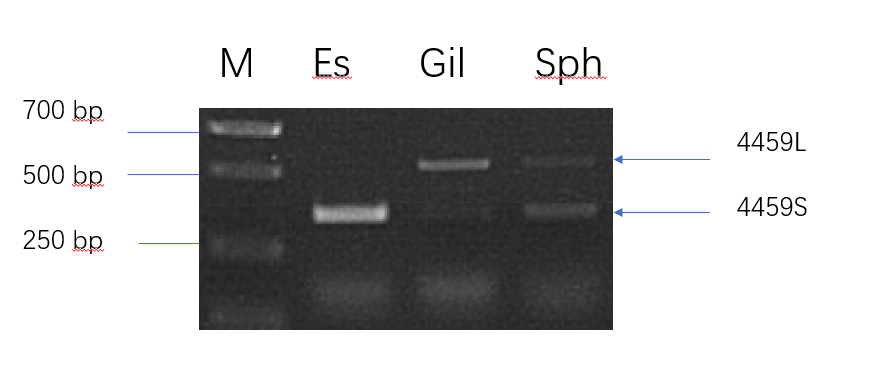


S2 Fig RT-PCR detection of transcripts 4459L and 4459-S in the eyestalk (Es), gill (Gil) and spermatophore (Sph) of the *F. merguienesis*
