# Supplementary material for: Functional and evolutionary implications from the molecular characterization of five spermatophore CHH/MIH/GIH genes in the shrimp Fenneropenaeus merguiensis
Source: PLoS One. 2018 Mar 19;13(3):e0193375. doi: 10.1371/journal.pone.0193375 (PMC5858750; doi:10.1371/journal.pone.0193375)
Supplement: S1 Table — (DOCX) [file pone.0193375.s003.docx]

S1 Table : Primers used in qRT-PCR studies and RT-PCR of transcripts 4459, 8101, 28020, 32710, 14056 and the control gene elongation factor (EF)

| Names | Sequence | Amplicon size (bp) |
| --- | --- | --- |
| 4459rtF | 5’-GGTTGTAGCAGTCCTCGCAG-3’ | 157 |
| 4459rtR | 5’-GGTTGTAGCAGTCCTCGCAG-3’ |  |
| 8101rtF | 5’-GGATGAGCGGAAAGTGGTTC -3’ | 153 |
| 8101rtR | 5’-CTTCTCGTTTCTTGCCGCTT -3’ |  |
| 28020rtF | 5’-TTCAGCAAGCGAGCGAACTT -3’ | 107 |
| 28020rtR | 5’-TCGCGAAACACGTTGTAGCA -3’ |  |
| 32710rtF1 | 5'-ATCGGCCCACAGACACTACA-3 | 171 |
| 32710rtR1 | 5’-TCGAGGCTCTCTTGCCTCTC -3 |  |
| 14056rtF | 5’-CGACTGATGGCATCTCCACG-3’ | 160 |
| 14056rtR | 5’-CGTAGATGTTCCGGTTGCCC-3’ |  |
| EFrtF | 5’-TGTTCCAGCGAGACAAGCCC -3’ | 252 |
| EFrtR | 5’-TGTAATCGGCATGGCCTGGG -3’ |  |
